# Development of a Competency Scale for Rehabilitation Professionals Supporting Kayoi-no-ba in Japan

**DOI:** 10.1298/ptr.E10334

**Published:** 2025-05-27

**Authors:** Mutsumi NAKAMURA, Yoshifumi URABE

**Affiliations:** 1 Department of Physical Therapy, Tohto University Faculty of Human Care at Makuhari, Japan; 2 Department of Rehabilitation Medicine, Ohashi Hospital, Japan

**Keywords:** Long-term care prevention, Rehabilitation professionals’ support, A competency scale, Kayoi-no-ba, Older residents

## Abstract

Objectives: Recently, rehabilitation professionals in Japan have become increasingly involved in Kayoi-no-ba for long-term care prevention. However, there are significant differences in the quality and content of professional rehabilitation support. This study aimed to develop a competency scale for rehabilitation professionals who support Kayoi-no-ba. Methods: A survey was mailed to rehabilitation supervisors at 440 facilities selected from a nationwide Internet search of facilities that implement the “community-based rehabilitation activity support project,” and rehabilitation professionals involved in supporting Kayoi-no-ba were asked to respond to the survey. The questionnaire was self-administered, and responses were sought via a 6-point scale for the 50 proposed competency items. For statistical analysis, exploratory factor analysis was conducted to examine the factor structure of the scale. Covariance structure analysis was used to assess model fit and to evaluate the reliability and internal consistency of the scale. Results: Among the 260 facilities that responded (59.1%), 607 participants provided valid responses. The final competency scale had a 3-factor structure (knowledge and attitude, ability to coordinate groups, and clinical practice skills) comprising 20 items. Model fit and internal consistency were good. Conclusions: Our findings indicate that rehabilitation professionals who support Kayoi-no-ba should have the attitude and knowledge of supporters, the ability to coordinate groups, and the clinical practice skills of rehabilitation therapists. This competency scale could be used as a guideline to improve the quality of support.

## Introduction

Japan has one of the world’s oldest populations. As a result, strategies to address the aging population have been implemented to promote health and prevent frailty. In 2013, a new insurance system for care prevention, the Comprehensive Project for Care Prevention and Daily Life Support (Comprehensive Project), was established. Japan has focused on the population approach as a major strategy for long-term care prevention and recommends the nationwide development of “Kayoi-no-ba”^1)^. The term Kayoi-no-ba broadly refers to activities focused on resident-centered frailty prevention through physical exercises, hobbies, or other activities^2)^. As an effect of Kayoi-no-ba, a lower risk of developing frailty has been reported in areas with higher rates of participation in Kayoi-no-ba^3)^. In addition, participation in Kayoi-no-ba has been shown to lower the risk of developing dementia^4,5)^ and tends to reduce future care costs^6)^.

In the Comprehensive Project, the “Community-based rehabilitation activity support project” has been newly established, and the involvement of rehabilitation professionals and others in Kayoi-no-ba is recommended^7)^. Therefore, the frequency with which rehabilitation professionals provide support for Kayoi-no-ba has been increasing year by year^8)^. On the other hand, it has been pointed out that there are significant individual and regional differences in the content and quality of the support provided by rehabilitation professionals^9)^. Rehabilitation professionals usually provide individual support in the medical and long-term care insurance fields and have relatively few opportunities to support groups; thus, many rehabilitation professionals are concerned about the content of support. Therefore, there is a need for concrete measures to implement effective support^9)^. However, there is little information on the content of physical therapists’ population-based practice (PBP)^10,11)^. A recent international study of physical therapist education programs reported low rates of content related to primary and secondary prevention, as well as marked variation across programs in health assessment and lifestyle-related topics^10)^. With the goal of implementing a safe, high-quality health promotion role for health professionals in the community, several studies have attempted to establish competency standards in the practice of health promotion and wellness activities as an educational program for health professionals^12–14)^. Therefore, this study attempted to develop a competency scale for rehabilitation professionals to develop their ability to perform their duties, utilizing knowledge, skills, attitudes, and values to meet the complex demands of uncertain situations when providing support to Kayoi-no-ba. The items for the scale were extracted from qualitative research, a draft item plan was created, and the respondents were asked to answer the questions. A factor analysis was conducted on the data obtained from the responses to create a scale, and the reliability and validity of the scale were verified. This scale is intended for rehabilitation professionals who are providing support to Kayoi-no-ba, and its analysis and results are meant to be used by the rehabilitation professionals themselves or by the lead administrator or head of their facility who has requested the support. Establishing a competency scale is expected to clarify the direction of the support provided by rehabilitation professionals and reduce differences among rehabilitators with respect to the content and quality of support^15)^. In addition, by referring to the items of the competency scale presented in this study, it is possible to review one’s own support methods and understand what items are lacking and what should be implemented. Furthermore, it is thought that the items can be utilized in teaching support methods as part of competency education. This study aimed to create a competency scale for rehabilitation professionals who support Kayoi-no-ba. The term “competencies” in this study was defined according to the Australian occupational therapy competency standards^16)^ as follows: an individual ability that integrates elements of knowledge, skills, values, and attitudes that are demonstrated to fulfill the role of a rehabilitation professional.

## Methods

### Participants

The target population consisted of rehabilitation professionals involved in supporting Kayoi-no-ba as part of long-term care prevention. The sample size for a survey should be 7 times the number of items in the survey and at least 100 persons^17)^. Therefore, assuming a mail survey collection rate of 40%^18)^, the sample size was set at 350 persons. The survey was sent to 440 facilities, assuming that an average of 2 persons per facility would respond. To ensure a sufficient sample size with minimal selection bias, we performed an unbiased search of the internet for hospitals and facilities throughout Japan related to the Long-Term Care Insurance Act that implemented the community-based rehabilitation activity support project. Accordingly, we identified 440 facilities that were selected in order of their search order. The 440 facilities covered 18 prefectures and 171 municipalities. A survey form was mailed to the rehabilitation department managers of the selected facilities, and rehabilitation professionals (physical, occupational, and speech-language-hearing therapist) involved in supporting Kayoi-no-ba were asked to respond. Because this scale was developed with the intention of being used by professionals with little experience as well as those with a great deal of experience, we did not limit the target population based on years of experience.

### Investigation

The survey was conducted by mail. The collection period was from February 21, 2023 to March 31, 2023. The survey was conducted using a self-administered, unmarked questionnaire regarding the basic attributes of the participants and proposed competency items to be acquired by rehabilitation professionals who provide support to Kayoi-no-ba. The basic attributes included sex, age, professional qualification, years of professional experience, and support experience. The proposed competency items comprised 50 items in a tentative scale version established from keywords identified from a previous study^19)^. In creating the scale, a pretest was conducted by 6 physical and occupational therapists experienced in providing support for community-based activities for older residents to review the plainness and validity of the text, and repeated revisions were made to the scale. Guidance on the tentative version of the scale was provided by 2 researchers specializing in medical health (a physical therapist and a dietitian) with experience in conducting research using the modified grounded theory approach to ensure the validity of the constructs. In the survey request, we explained as follows: “Based on the responses we receive, we plan to examine the reliability and validity of this rating scale and determine the items for the competency scale.” Respondents were asked, “Please select the following selection criteria for what you implement when you provide support to Kayoi-no-ba, and place a circle around the number that is closest to you.” The respondents were asked to score their answers on a 6-point scale (1, not at all applicable; 2, not applicable; 3, not somewhat applicable; 4, somewhat applicable; 5, applicable; and 6, very applicable).

### Analysis

The obtained data were first reviewed for the presence or absence of ceiling or floor effects. Items that had ceiling effects (mean + standard deviation > 6) and floor effects (mean − standard deviation < 1) were excluded^20)^. Next, we conducted an item-total correlation analysis to confirm the correlation coefficient between each item and the total score (r ≥ 0.7), followed by an exploratory factor analysis to examine the factor structure of the scale. In determining the names of the factors, we found commonalities among each of the extracted items, which represent the characteristics of each factor as a necessary capability for passive place support. Subsequently, the model fit of the obtained factor structure was confirmed using a covariance structure analysis, followed by verification of the construct validity of the created model. The goodness of fit of the model was determined using the goodness of fit index (GFI), adjusted goodness-of-fit index (AGFI), comparative fit index (CFI), and residuals. Moreover, we assessed the model using the root mean square error of approximation (RMSEA)^21)^. To assess the reliability of the scale, Cronbach’s *α* coefficient was calculated for the total score and the scores for each factor; moreover, internal consistency was verified. Here, the reliability criterion was a Cronbach’s *α* coefficient of ≥0.70, which is generally considered desirable in scale creation^21,22)^. The determination of the factor names was validated with 1 researcher and 2 collaborators (2 physical therapists and 1 occupational therapist) with extensive experience and expertise in providing support to Kayoi-no-ba. Statistical analyses were performed using IBM SPSS Statistics (ver. 27 IBM Japan, Tokyo, Japan) and IBM Amos SPSS Statistics (ver. 29 IBM Japan, Tokyo, Japan), with the significance level set at 5%.

### Ethical considerations

The Tohto University Research Ethics Review Committee approved this study (approval number: R0409). The participants received explanations regarding the study in the research cooperation request form, which stated the study purpose, methodology, ethical considerations, voluntary participation in the research, and the lack of disadvantages for declining the research. Returning the questionnaire was considered an agreement to participate in the research.

## Results

Among the 440 facilities surveyed, 260 responded (response rate 59.1%), with 609 people from these facilities responding. The average (standard deviation) number of participants per facility was 2.3 (1.6) (range 0–10). Among them, responses from 607 people, excluding 2 who did not respond, were considered valid. Table 1 presents the participants’ characteristics. The participants included 425 males (70.0%) and 180 females (29.7%), with a mean (standard deviation) age of 39.0 (8.3) years.

**Table 1.** Characteristics of the participants and support status of Kayoi-no-ba

		N or mean	% or SD
Characteristics of the participants			
Sex	Male	425	70.0%
	Female	180	29.7%
	Unanswered	2	0.3%
Age (years)		39.0	8.3
Professional qualification	Physical therapist	417	68.7%
	Occupational therapist	157	25.9%
	Speech-language-hearing therapist	28	4.6%
	Unanswered	5	0.8%
Professional experience (years)		15.0	4.4
Support experience (years)		8.0	3.8

N = 607. Age, years of experience, and years of experience supporting Kayoi-no-ba are shown as means (SD)

SD, standard deviation

Among the 607 participants, we excluded data from 10 participants with missing responses to the 50 items; accordingly, we included data from the remaining 597 participants in the analysis. We calculated the mean and standard deviation of each item to confirm the ceiling and floor effects of the proposed items. Ceiling effects were observed in 5 items; 8, 11, 12, 17, and 20. No floor effect was observed. These ceiling effect items are important when providing support to Kayoi-no-ba. However, they were excluded due to their uneven distribution, which impeded the extraction of their characteristics. The correlation coefficient showed the lowest value for item 2 (r = 0.277) and the highest value for item 50 (r = 0.729); however, there was no significant correlation for any of the items. There was a strong correlation between items 21 and 22 (correlation coefficient = 0.728) and between items 49 and 50 (0.744). Accordingly, we adopted items that were more comprehensive and clearer in capturing the semantic content of the questions, leading to the subsequent exclusion of items 22 and 49. Among the 50 questionnaire items, 43 were analyzed, excluding 5 items with ceiling items and 2 items with strong inter-item correlations. Exploratory factor analysis (generalized least squares and varimax rotation) was performed. Regarding commonality, we set a factor loading of ≥0.400 as the standard^11)^ and excluded items with lower loadings, items with high loadings (≥0.400) on multiple factors, and items with a correlation coefficient ≥0.700. This analysis was repeated (Table 2). Finally, a 3-factor structure comprising 20 items was estimated (Table 3).

**Table 2.** List of excluded items

Reason for exclusion	Factor loadings	Item	
Ceiling effect		Q8	Adjusting my day job to be involved in long-term care prevention project
		Q11	Be easily spoken to and treated in an accepting atmosphere
		Q12	Provide participants with easy-to-understand explanations without using too many technical terms
		Q17	Responding sincerely to participants' requests for advice and requests
		Q20	Praise and approval for activities and participants
Inter-item correlation		Q15	Provide support that is beneficial to participants, not self-satisfying
		Q16	Be close to participants' thoughts and feelings
		Q21	Be aware that the activity is participant-driven, and elicit their initiative
		Q22	Have a viewpoint to support independence
		Q40	Build face-to-face relationships to facilitate information sharing with other professions
		Q42	Understand the expertise and roles of other professions
		Q49	Provide support from a continuous perspective, not just on the spot
Factor analysis of exploration	<0.40	Q2	Seeking advice from experienced (senior) staff to resolve concerns about support
		Q9	Provide support that makes you feel a sense of fulfillment and accomplishment
		Q39	When providing support, they are aware of the effects and results
	>0.40 for 2 factors	Q1	Understand the role required of rehabilitation professionals
		Q3	Learn about local characteristics and social resources
		Q6	Understand municipal policies, welfare plans, and projects
		Q10	Be passionate about support
		Q24	Provide professional advice
		Q27	Advice on gymnastics and exercise
		Q30	Provide advice with consideration to physical condition, blood pressure, and health status
		Q31	Check the status of infection control measures during activities and provide advice
		Q33	Explain to participants the purpose of the support
		Q38	Notice the characteristics of the group and the diversity of its members
		Q43	Become aware of issues and problems in local activities and businesses
		Q44	Sharing information between rehabilitation professionals about support content, issues, solutions, etc.
		Q45	Ability to be aware of linkages with other project (service project, comprehensive support project, etc.)
		Q48	Utilize support experience for subsequent advice and support
		Q50	Provide support with an awareness of community development

**Table 3.** Exploratory factor analysis results

Item		Factor		
		1st factor: knowledge and attitude	2nd factor: ability to coordinate groups	3rd factor: clinical practice skills
Q4	Have an inquisitive mind about the prevention of long-term frailty and self-study	**0.568**	0.376	0.139
Q5	Refer to the support methods of experienced rehabilitation professionals	**0.450**	0.222	0.127
Q7	Prepare in advance by doing research to eliminate any anxiety about support	**0.575**	0.213	0.117
Q13	Be aware of the distance from participants	**0.698**	0.168	0.137
Q14	Draw out opinions without imposing your own ideas on participants	**0.719**	0.261	0.118
Q18	Be open to participants' opinions and feelings	**0.716**	0.211	0.134
Q19	Motivate participants	**0.739**	0.316	0.167
Q23	Do not give excessive support or advice, and do not interfere too much	**0.550**	0.295	0.177
Q25	Provide information on nursing prevention of long-term frailty to participants	**0.510**	0.344	0.329
Q29	Perform risk management in the environment of venues and activity locations	**0.420**	0.336	0.329
Q32	Utilizing knowledge and experience gained at hospitals and nursing care facilities for support	**0.529**	0.141	0.323
Q34	Sharing participants' impressions and opinions with the entire group	0.331	**0.587**	0.226
Q35	Advise on the content and methods of group activities and operational issues	0.207	**0.701**	0.299
Q36	Consult with the group leader and give advice	0.269	**0.647**	0.258
Q37	Visit the places you frequent and find out what the daily routine is like	0.207	**0.466**	0.052
Q41	Share information with other professions and institutions	0.355	**0.562**	0.112
Q46	Provide advice and guidance to rehabilitation professionals with little support experience	0.295	**0.619**	0.156
Q47	Propose issues and solutions regarding projects and measures to business owners	0.179	**0.813**	0.156
Q26	Assess and give advice on functional impairment such as pain and limited range of motion	0.143	0.188	**0.815**
Q28	Advise participants on their movements, posture, and gait	0.228	0.253	**0.720**
Cronbach’s *α* all 0.922		0.893	0.862	0.799

Factor extraction method; generalized least squares method, rotation method; varimax rotation method

Items in bold indicate factor loadings with an absolute value of 0.400 or higher

Regarding the interpretation of each factor, the 1st factor comprised 11 items; among them, items 4, 5, 7, 13, 14, 18, 19, 23, 25, 29, and 32, which were related to preparation, knowledge, and attitude, had high loadings. Accordingly, the 1st factor was called “knowledge and attitude.” The 2nd factor comprised 7 items; among them, items 34, 35, 36, 37, 41, 46, and 47, which were related to working with groups, had high loadings. Accordingly, the 2nd factor was designated “ability to coordinate groups.” The 3rd factor comprised 2 items and was called “clinical practice skills,” since items 26 and 28, which were related to assessment and treatment skills, had high loadings.

Covariance structure analysis was conducted to verify the goodness of fit of the 3-factor, 20-item model obtained through factor analysis. The results were as follows: GFI = 0.896, AGFI = 0.869, CFI = 0.909, and RMSEA = 0.072, which indicated acceptable levels of compliance^21)^.

To confirm internal consistency, Cronbach’s *α* coefficient for all 20 items was calculated and found to be 0.922. Further, we calculated the *α* coefficient for each factor and found that it was 0.893, 0.862, and 0.799 for the 1st, 2nd, and 3rd factors, respectively (Table 3).

## Discussion

This study established a competency scale for rehabilitation professionals who provide support to Kayoi-no-ba by a structural analysis of covariance. Moreover, factor structure analysis of the scale resulted in a 3-factor, 20-item structure (knowledge and attitude, ability to coordinate groups, and clinical practice skills).

Factor 1, “knowledge and attitude,” comprised 11 items. These included items related to preparation and knowledge acquisition to provide effective support, such as “have an inquisitive mind about prevention of long-term frailty and self-study” (I-4) and “refer to the support methods of experienced rehabilitation professionals” (I-5). Furthermore, it included items regarding the attitude of the rehabilitator as a supporter, including “be aware of distance from participants” (I-13) and “draw out opinions without imposing your own ideas on participants” (I-14). Rehabilitation professionals have been involved in secondary and tertiary prevention, often targeting people with physical disabilities. In recent years, rehabilitation professionals have been expected to play an active role in the field of health promotion and prevention^23)^. However, there is a lack of knowledge and expertise in PBP for physical therapists, which has not traditionally been viewed as a role for them^11,24)^. Current World Physiotherapy (WPT) states that physical therapists need specific competencies to consistently practice health promotion^13)^. In PBP, knowledge and attitudes will lead to effective supportive practices and policy development^25)^.

This study focused on PBP by rehabilitation professionals in Kayoi-no-ba, mainly for the purpose of preventing the progression of age-related frailty in the older adult population. Hospitals and long-term care facilities often use an individual approach, which differs in many respects from PBP in Kayoi-no-ba. Rehabilitators who provide support to Kayoi-no-ba need to be aware that general knowledge that contributes to health promotion, not just physical activity, is required. In the field of health promotion and prevention, knowledge is required not only about physical activity and exercise but also about nutrition, smoking cessation, sleep, stress management, and behavioral changes^26)^. However, the rehabilitation profession lacks knowledge of these topics, and it has been suggested that opportunities for learning be provided during the pre- and postgraduate educational processes^26–28)^. As described above, rehabilitation professionals who provide support to Kayoi-no-ba are required to have a broad range of knowledge in the field of health promotion and prevention, as well as an attitude suitable for PBP.

Factor 2, “ability to coordinate groups,” comprised 7 items related to the ability to coordinate the entire group rather than each individual, including “sharing participants’ impressions and opinions with the entire group” (I-34) and “advise on the content and methods of group activities and operational issues” (I-35).

Group activities led by residents have many problems, such as getting stuck in a rut and the issues related to relationships among members^29)^. Rehabilitation professionals are required to provide advice and coordination to ensure that group activities continue and expand autonomously when providing support to Kayoi-no-ba. In addition, items such as “Share information with other professions and institutions (I-41)” and “Propose issues and solutions regarding projects and measures to business owners (I-47)” are important. To practice prevention and health promotion in the community, collaboration with other organizations and multiple professions beyond the facility to which one belongs is required^10)^. Continuing to practice a population approach in the community will further develop these abilities^10)^.

Factor 3, “clinical practice skills,” comprised 2 items, that is, “assess and give advice on functional impairment such as pain and limited range of motion” (I-26) and “advise participants on their movements, posture, and gait” (I-28), which are related to the assessment and treatment skills traditionally used by rehabilitation professionals. Regarding the prevention of frailty, rehabilitation professionals are expected to promote initiatives that contribute to independence and support and strengthen functioning to prevent frailty^1)^. Furthermore, it is important to work with older adults who have impaired life functions in a well-balanced manner across the respective elements of “body functions and structure,” “activities,” and “participation” in the International Classification of Functioning, Disability and Health (ICF)^30)^. Regular involvement of rehabilitation professionals and others in Kayoi-no-ba could facilitate guidance on exercise methods that facilitate continuous participation despite physical disabilities and joint pain, provide guidance to caretakers on how to handle individuals with dementia, conduct periodic physical fitness measurements, and address other matters related to exercise functions. Moreover, it could facilitate the participation of individuals requiring Kayoi-no-ba^27)^.

In its model core curriculum for physical therapy education, the Japan Physical Therapy Association lists the following 6 basic qualities and abilities required of physical therapists: (1) professionalism, (2) knowledge and skills in physical therapy, (3) evidence-based problem solving skills, (4) quality assurance and safety management, (5) encouragement of lifelong learning, and (6) communication skills^31)^. These qualities and abilities were also included as essential for supporters of Kayoi-no-ba. Magnusson et al^12)^. conducted a Delphi study to develop population health, prevention, health promotion, and wellness competencies and coordinate group competencies for the professional education of physical therapists. Of the 25 competencies identified, 18 were selected in the category of “clinical and community preventive services and health promotion.” Among them were “communicate prevention and health promotion information in a way that recognizes and respects clients’ values, priorities, and communication needs” and “design evidence-based injury prevention programs to make homes, communities, schools, and worksites safer” in this study. Also, “function as a member of an inter-professional team of health professionals, community health workers, public health professionals, and others to reduce disease risk and improve health among individuals and populations” and “establish and foster client-centered and inter-professional collaborations that empower individuals and populations” were included in Factor 2 under “ability to coordinate groups” in this study.

Taken together, the constructs of the subscales of the 20 items and the 3 factors extracted in this study could be explained based on the elements extracted through literature synthesis, which confirmed their validity.

The scale was confirmed to be reliable and valid; furthermore, it is expected to be used to evaluate the practical ability of rehabilitation professionals who provide support to Kayoi-no-ba and can be utilized for step-by-step education according to their own abilities and weaknesses. Additionally, reference to the scale items could inform the provision of support by rehabilitation professionals for Kayoi-no-ba as well as reduce differences among rehabilitators in terms of the content and quality of support. The scale could help supporters check and reflect on the content of their support.

This study had 3 limitations. First, there are differences in the characteristics of community rehabilitation support projects across municipalities, and the roles required of rehabilitation professionals may differ according to these characteristics. Therefore, it is conceivable that the items emphasized in the present study may differ regionally. Secondly, since this study did not limit the number of years of experience among the subjects, it is possible that variation in responses could be observed. Thirdly, in this study, we did not the evaluate test–retest reliability, although we did verify it through internal consistency as an evaluation of reliability. However, since this study covered the entire country and diverse project entities, our competency scale may be generalizable across geographical regions. In the future, we would like to conduct a survey using the competency scale to examine whether it can be used in clinical practice. Additionally, we would like to examine educational methods for enhancing the practical skills of rehabilitation professionals who provide support to Kayoi-no-ba.

## Conclusions

This study developed a 20-item competency scale with 3 subscales for rehabilitation professionals who support Kayoi-no-ba, which demonstrated good reliability and validity. Our findings indicated that these professionals should have the attitude and knowledge of supporters, mediation skills, and the clinical skills of rehabilitation therapists.

## Acknowledgments

We would like to acknowledge the participants of this study.

## Funding

This study was supported by a Grant-in-Aid for Scientific Research (KAKENHI No.20K19457) from the Japan Society for the Promotion of Science.

## Conflict of Interest

The authors declare no conflicts of interest.
